# A nomogram for predicting the HER2 status of circulating tumor cells and survival analysis in HER2-negative breast cancer

**DOI:** 10.3389/fonc.2022.943800

**Published:** 2022-12-21

**Authors:** Yuqin Yang, Liudan Li, Wenjing Tian, Zhen Qiao, Qi Qin, Liqian Su, Peiqiu Li, Weirong Chen, Hong Zhao

**Affiliations:** ^1^ The Cancer Center of The Fifth Affiliated Hospital of Sun Yat-Sen University, Zhuhai, Guangdong, China; ^2^ Department of Pathology, School of Basic Medical Science, Southern Medical University, Guangzhou, Guangdong, China; ^3^ Guangdong Provincial Key Laboratory of Biomedical Imaging, The Fifth Affiliated Hospital, Sun Yat-sen University, Zhuhai, Guangdong, China; ^4^ Department of Breast Surgery, Zhuhai Maternity and Child Health Hospital, Zhuhai, Guangdong, China; ^5^ Department of Medical Oncology, The Second Affiliated Hospital of Hainan Medical University, Haikou, Hainan, China; ^6^ Precision Medicine Center of Harbin Medical University Cancer Hospital, Harbin, Heilongjiang, China; ^7^ Department of Nephrology, The Fifth Hospital Affiliated of Sun Yat-sen University Zhuhai, Guangdong, China

**Keywords:** breast cancer, circulating tumor cells, HER2, nomogram, de ritis ratio, uric acid, survival analysis

## Abstract

**Background:**

In breast cancer patients with HER2-negative tumors (tHER2-), HER2-positive CTCs (cHER2+) were associated with promising efficacy of HER2-targeted therapy, but controversy has persisted over its prognostic effect. We developed a model including clinicopathologic parameters/blood test variables to predict cHER2 status and evaluated the prognostic value of cHER2+ in tHER2- patients.

**Methods:**

cHER2+ was detected, blood test results and clinicopathological characteristics were combined, and a nomogram was constructed to predict cHER2 status in tHER2- patients according to logistic regression analysis. The nomogram was evaluated by C-index values and calibration curve. Kaplan–Meier curves, log-rank tests, and Cox regression analyses were performed to evaluate the prognostic value of cHER2 status.

**Results:**

TNM stage, white blood cells (WBCs), neutrophils (NEUs), uric acid (UA), De Ritis ratio [aspartate transaminase (AST)/alanine transaminase (ALT)], and high-density lipoprotein (HDL) were found to be associated with cHER2 status in tHER2- patients in univariate logistic regression analysis, in which UA and De Ritis ratio remained significant in multivariate logistic regression analysis. A model combining these six variables was constructed, the C-index was 0.745 (95% CI: 0.630–0.860), and the calibration curve presented a perfect predictive consistency. In survival analysis, patients of the subgroups “with cHER2+/UA-low” (*p* = 0.015) and “with cHER2+/De Ritis ratio – high” (*p* = 0.006) had a significantly decreased disease-free survival (DFS).

**Conclusions:**

Our nomogram, based on TNM stage, WBC, NEU, UA, De Ritis ratio, and HDL, may excellently predict the cHER2 status of tHER2- patients. Incorporation with UA and De Ritis ratio may enhance the prognostic value of cHER2 status.

## Introduction

As the most common cancer in women, breast cancer accounts for almost one-third of new cancer diagnoses and one-seventh of cancer-related deaths in the US (1). The combination of surgery and systemic therapy has become the standard treatment strategy for breast cancer, especially the application of HER2-targeted therapy (2). The poor prognosis of HER2+ breast cancer patients can be significantly improved by prolonging disease-free survival (DFS) and overall survival (OS) (3). However, due to inefficiency, HER2-targeted therapies are usually not applied to patients with HER2-negative tumors (tHER2−).

Breast cancer is well known to be a heterogeneous disease (4), and the HER2 status assessed by tissues from surgery or needle biopsy may not represent the heterogeneity of breast cancer (5). Detached from the primary or metastatic lesion, circulating tumor cells (CTCs) are disseminated into the blood and consist of the most aggressive subset of cancer cells (6) and were found to be a negative prognostic marker in early and advanced breast cancer (7, 8). The expression of HER2 on CTCs has been detected, and discordance in HER2 status between tumors *in situ* and CTCs was found in up to 50% of patients (9–11). However, when interpreting the results of prognostic values, controversy has persisted over the HER2 status on CTCs (12–15). However, a surprising consensus came by studies showing that HER2+ CTCs offer the chance of successfully used HER2-targeted therapy in tHER2- patients (12, 16–18). Meng et al. showed that three achieved partial or complete response after nine advanced breast cancer patients with tHER2−/cHER2+ who received Herceptin-containing schedules (16). Nevertheless, due to the cost and availability, HER2 expression in CTCs was not detected in the vast majority of tHER2- patients.

An overall consensus came from the observation that for HER2+ breast cancer, the earlier the anti-HER2-targeted therapy is administered, the better (2). Thus, if a predictive model that uses routine clinical test results/clinicopathological parameters to predict patients’ cHER2 status is developed, it could be used to screen suitable patients and detect the expression of HER2 in CTCs. This model would have great clinical value for improving the survival of these patients, as anti-HER2 agents could be administered early. In previous studies, hormone receptor (HR) status, tumor subtype, white blood cells (WBCs), neutrophils (NEUs), and monocytes were demonstrated to be correlated with the frequency of CTCs (19, 20). In our present study, the results suggested that there was a correlation between clinicopathologic parameters/blood test variables and cHER2 status, and these parameters/variables may predict the cHER2 status of tHER2- patients.

Herein, we trained a model combining baseline blood test results, including routine blood tests and blood biochemical tests, and clinicopathological characteristics. With this model, we aimed to distinguish the cHER2 status of tHER2- breast cancer patients, which can guide more personalized therapeutic strategies for tHER2- patients. Moreover, we analyzed the prognostic impact of cHER2 status in tHER2- patients.

## Methods

### Patient population and data collection

In the current study, patients diagnosed as breast cancer at Zhuhai Maternity and Child Health Hospital between November 2017 and November 2018 were recruited. The inclusion criteria were as follows (1): agree to participate in the trials and provide the written consent; (2) age from 18 to 70; (3) able to follow the schedule; (4) no other history of a malignant tumor; and (5) TNM stage I–IV (pathological staging) breast cancer. Exclusion criteria included (1) pregnant or breastfeeding; (2) participating other clinical trial at the same time; (3) with clear surgery contraindications; and (4) other reasons not suitable for participation.

Clinicopathological parameters and blood test variables were collected for all patients, including age at diagnosis, pathological type, TNM stage, tumor size, nodal status, estrogen receptor (ER) status, progesterone receptor (PR) status, Ki67, and blood test variables (white blood cells (WBC), lymphocytes (LY), monocytes (MONO), neutrophils (NEU), platelet (PLT), De Ritis ratio [aspartate transaminase (AST)/alanine transaminase (ALT)], triglyceride (TG), total cholesterol (TC), low-density lipoprotein (LDL), high-density lipoprotein (HDL), uric acid (UA), creatinine (CREA), and urea (UREA)).

Age, Ki67, and blood test parameters were analyzed as continuous variables with original data. Pathological type was divided into ductal carcinoma and other. TNM stage was grouped as I, II, III, and IV. The tumor size was divided into T1, T2, T3, and T4. For the nodal status, all cases were classified into four groups: N0, N1, N2, and N3. ER and PR were grouped into positive and negative. (The UA and De Ritis ratio was divided into low/high according to the median in Cox analysis and survival analysis.)

After the initiation of a new therapy, the patients were followed at 3–5 weeks and then 3–6 months, which varied depending on the treatment plans and patient conditions. The primary and secondary outcomes were recurrence/relapse and death.

According to the American Society of Clinical Oncology/College of American Pathologists Clinical Practice Guideline, no/weak (1+) immunohistochemical staining on the cell membrane or a negative result from the fluorescence *in situ* hybridization test when the cell membrane presented moderate (2+) staining was defined as negative HER2 expression (21). Notably, HER2 status was based on the samples from surgical resection.

The present study was reviewed and approved by the Ethics Committee of Zhuhai Maternity and Child Health Hospital (approval number: [2017] ICE-16) and registered in the Center of Chinese Clinical Trial Registry (registration number: ChiCTR1800015712). Written informed consent was gained from all participants.

### CTC enumeration and cHER2 detection

The enrichment and enumeration of HER2-positive CTCs using the Liquid Biopsy System (Livzon Gene Diagnostics Ltd., Zhuhai, Guangdong, China) was essentially performed as described in our previous study (22).

**Figure 1:**
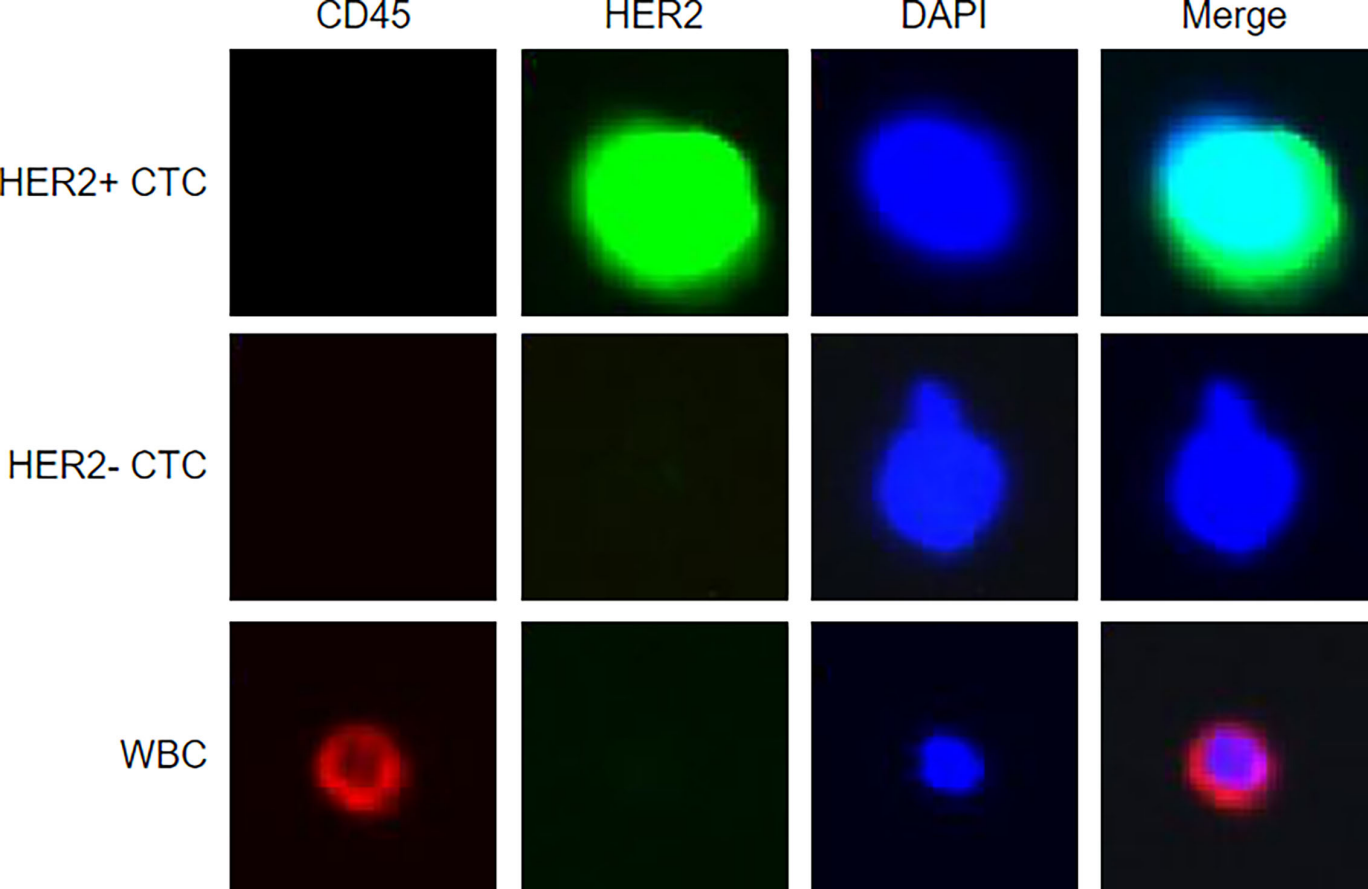
Representative figures of HER2+/HER2- circulating tumor cells and white blood cells.

In brief, 4 ml peripheral blood was drawn (right after being diagnosed with BC by biopsy), stabilized with fixative, and then lysed. CTCs were captured by using biotinylated epithelial–mesenchymal transition cocktails and streptavidin beads. Anti-HER2 monoclonal antibodies (HER2-if488) and anti-CD45 monoclonal antibodies (CD45-if647) were used to counterstain the CK-positive CTCs. Nucleated cells that expressed HER2 and did not express CD45 were defined as HER2+ CTCs. Representative images of HER2+/HER2- CTCs and WBCs are shown in **Figure 1**. The identification and counting of CTCs were performed by Livzon Gene Diagnostics Ltd. (Zhuhai, China) independently.

### Statistical analysis

The chi-square test and Mann–Whitney U test were utilized to analyze the categorical variables and continuous variables of the clinicopathological characteristics, respectively. A *p* value lower than 0.05 was considered to be significant. Potential predictive factors for HER2+ CTCs were screened out *via* logistic regression analyses. All *p* values were two-sided. In univariate logistic regression analysis, a *p* of 0.1 was considered significant to avoid excluding potential related factors, and in multivariate logistic regression analysis, *p* was set to be lower than 0.05. Based on the results of univariate logistic regression analysis, a nomogram was constructed and validated by multiple methods (C-index and calibration curve). The prognostic effect of the HER2 status of CTCs in patients was visualized using Kaplan–Meier curves. Cox regression analyses were used to identify variables for survival in patients (presented as hazard ratios [HRs] with 95% confidence intervals [CI]). Statistical analyses of the current study were performed using SPSS 22 (IBM, Armonk, NY, US) and R software (Institute for Statistics and Mathematics, Vienna, Austria).

## Results

### Patient characteristics

During this prospective clinical trial, 219 participants (consisting of 52 TNM stage I, 124 stage II, 40 stage III, 3 stage IV) were included. Because of the small cohort of TNM stage IV which may lead to great bias, all the three patients were excluded. In the present study, 216 patients with early breast cancer were analyzed, comprising 148 with HER2- primary tumor and 68 with HER2+.

**Figure 2:**
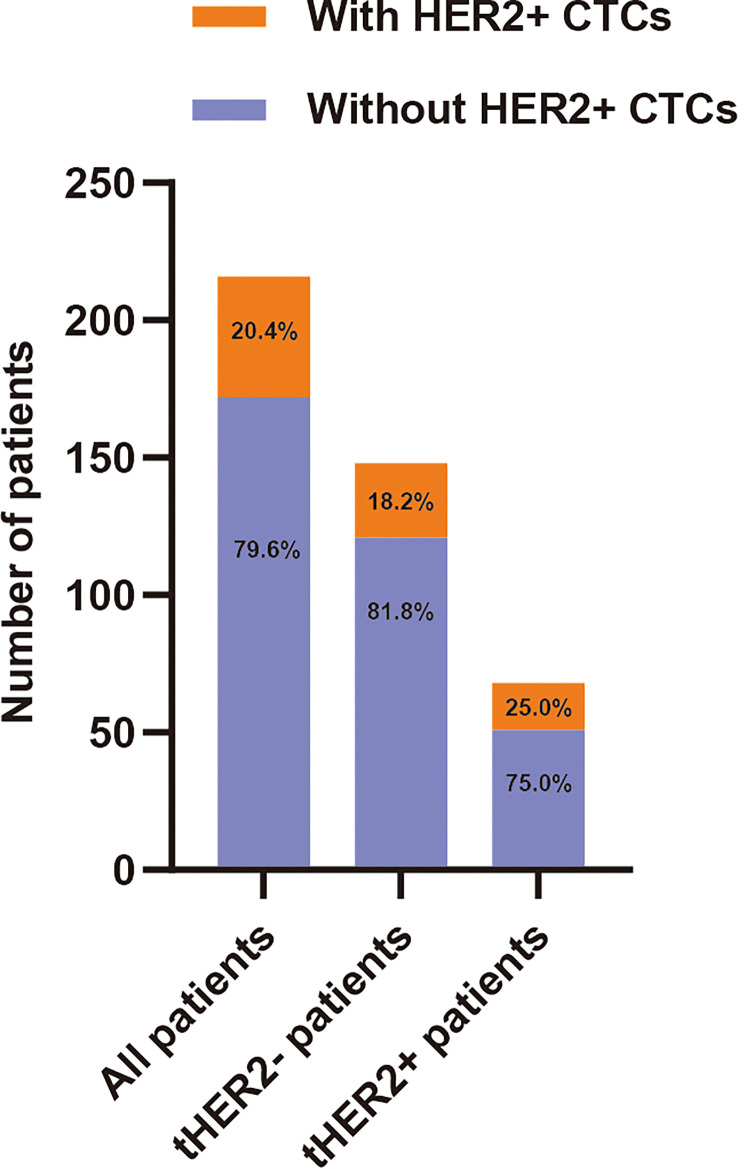
Distribution of HER2+ circulating tumor cells of patients with early breast cancer.

The frequency of CTCs detected in all 216 patients ranged from 0 to 39 (median: 1). HER2+ CTCs were observed in 44 patients (20.4%). Among the 44 cHER2+ patients, 27 (18.2%) and 17 (25.0%) patients in the tHER2- and tHER2+ groups, respectively, had one or more HER2+ CTCs (**Figure 2**). The detailed characteristics are shown in **Table 1**.

**Table 1:** Clinicopathologic parameters of 216 early breast cancer patients.

Variables	All patients (N = 216)	Patients with HER2- primary tumor (N = 148)	Patients with HER2+ primary tumor (N = 68)
Age, median (IQR)	46 (39, 52)	46 (39, 52)	47 (39, 50)
Pathological type, n (%) Ductal Other	194 (89.8) 22 (10.2)	137 (92.6) 11 (7.4)	57 (83.8) 11 (16.2)
TNM stage, n (%) I II III	52 (24.1) 124 (57.4) 40 (18.5)	35 (23.6) 82 (55.4) 31 (21.0)	17 (25.0) 42 (61.8) 9 (13.2)
Tumor size, n (%) T1 T2 T3 T4	68 (31.5) 130 (60.2) 13 (6.0) 5 (2.3)	47 (31.8) 87 (58.8) 9 (6.0) 5 (3.4)	21 (30.9) 43 (63.2) 4 (5.9) 0 (0.0)
Nodal status, n (%) N0 N1 N2 N3	116 (53.7) 65 (30.1) 21 (9.7) 14 (6.5)	78 (52.7) 42 (28.4) 16 (10.8) 12 (8.1)	38 (55.9) 23 (33.8) 5 (7.4) 2 (2.9)
ER status, n (%) Negative Positive	55 (25.5) 161 (74.5)	27 (18.2) 121 (81.8)	28 (41.2) 40 (58.8)
PR status, n (%) Negative Positive	68 (31.5) 148 (68.5)	35 (23.6) 113 (76.4)	33 (48.5) 35 (51.5)
Ki67, median (IQR)	0.20 (0.10, 0.40)	0.20 (0.10, 0.40)	0.20 (0.10, 0.40)

### Association between clinicopathological parameters and cHER2 status in HER2-/+ primary tumors

As shown in **Table 2**, the associations between clinicopathological characteristics, including age at diagnosis, pathological type, TNM stage, tumor size, nodal stage, ER status, and Ki67 and cHER2 status, were calculated.

**Table 2:** Association between clinicopathologic parameters and HER2+ circulating tumor cells status.

Variables	HER2- primary tumor (N = 148)		*p-value*	HER2+ primary tumor (N = 68)		*p-value*
	Without HER2+ CTC (N = 121)	With HER2+ CTC (N = 27)		Without HER2+ CTC (N = 51)	With HER2+ CTC (N = 17)	
Age, median (IQR)	47 (39, 53)	45 (38, 48)	0.211	48 (39, 51)	43 (38, 48)	0.316
Pathological type, n (%) Ductal Other	113 (93.4) 8 (6.6)	24 (88.9) 3 (11.1)	0.422	50 (98.1) 1 (1.9)	17 (100.0) 0 (0.0)	1.000
TNM stage, n (%) I II III	24 (19.8) 69 (57.0) 28 (23.2)	11 (40.7) 13 (48.2) 3 (11.1)	0.021*	11 (21.6) 33 (64.7) 7 (13.7)	6 (35.3) 9 (52.9) 2 (11.8)	0.361
Tumor size, n (%) T1 T2 T3 T4	36 (29.8) 75 (62.0) 6 (5.0) 4 (3.2)	11 (40.7) 12 (44.4) 3 (11.1) 1 (3.8)	0.784	16 (31.4) 32 (62.7) 3 (5.9) 0 (0.0)	5 (29.4) 11 (64.7) 1 (5.9) 0 (0.0)	0.900
Nodal status, n (%) N0 N1 N2 N3	62 (51.2) 35 (28.9) 13 (10.7) 11 (9.2)	16 (59.3) 7 (25.9) 3 (11.1) 1 (3.7)	0.361	28 (54.9) 18 (35.3) 4 (7.8) 1 (2.0)	10 (58.9) 5 (29.3) 1 (5.9) 1 (5.9)	0.927
ER status, n (%) Negative Positive	25 (20.7) 96 (79.3)	2 (7.4) 25 (92.6)	0.166	22 (43.1) 29 (56.9)	6 (35.3) 11 (64.7)	0.569
PR status, n (%) Negative Positive	31 (25.6) 90 (74.4)	4 (14.8) 23 (85.2)	0.232	26 (51.0) 25 (49.0)	7 (41.2) 10 (58.8)	0.484
Ki67, median (IQR)	0.20 (0.10, 0.40)	0.20 (0.08, 0.30)	0.101	0.20 (0.10, 0.40)	0.25 (0.10, 0.40)	0.780

**Figure 3:**
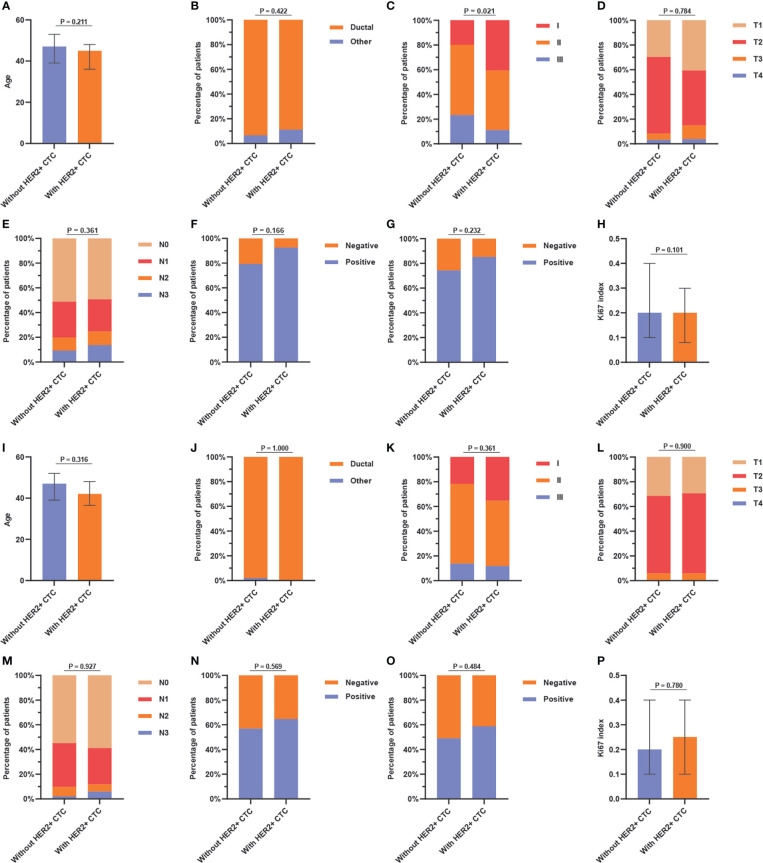
Association between **(A)** age, **(B)** pathological type, **(C)** TNM stage, **(D)** tumor size, **(E)** nodal status, **(F)** ER status, **(G)** PR status, and **(H)** Ki67 index and HER2+ circulating tumor cells status in tHER2- patients, and the association between **(I)** age, **(J)** pathological type, **(K)** TNM stage, **(L)** tumor size, **(M)** nodal status, **(N)** ER status, **(O)** PR status, and **(P)** Ki67 index and HER2+ circulating tumor cells status in tHER2+ patients. Note: age and Ki67 index present in median with the interquartile range.

In the tHER2- group, cHER2+ patients tended to have a lower age, lower percentage of ductal cancer, and lower expression of Ki67 but a higher expression of ER and PR (p = 0.211, 0.422, 0.101, 0.166, and 0.232, respectively). Patients without HER2+ CTCs had a higher TNM stage, larger tumor size, and more nodal involvement (*p* = 0.021, 0.784, and 0.361, respectively). However, no statistically significant differences were found in the clinicopathologic parameters above except for TNM stage (**Figures 3A-H**).

In the tHER2+ group, no significant associations were found between clinicopathologic parameters and cHER2 status in patients with tHER2+. However, tHER2+ patients with HER2+ CTCs had a tendency to be younger and earlier in disease (lower TNM stage) (p = 0.316 and 0.361, respectively). Additionally, higher expression of ER, PR, and Ki67 but similar trends for pathological type, tumor size, and nodal involvement were found in cHER2+ patients compared with those without cHER2+ CTCs (*p* = 0.569, 0.484, 0.780, 1.000, 0.900, and 0.927, respectively) (**Figures 3I-P**).

### Independent predictive factors for cHER2+ in tHER2-

**Figure 4:**
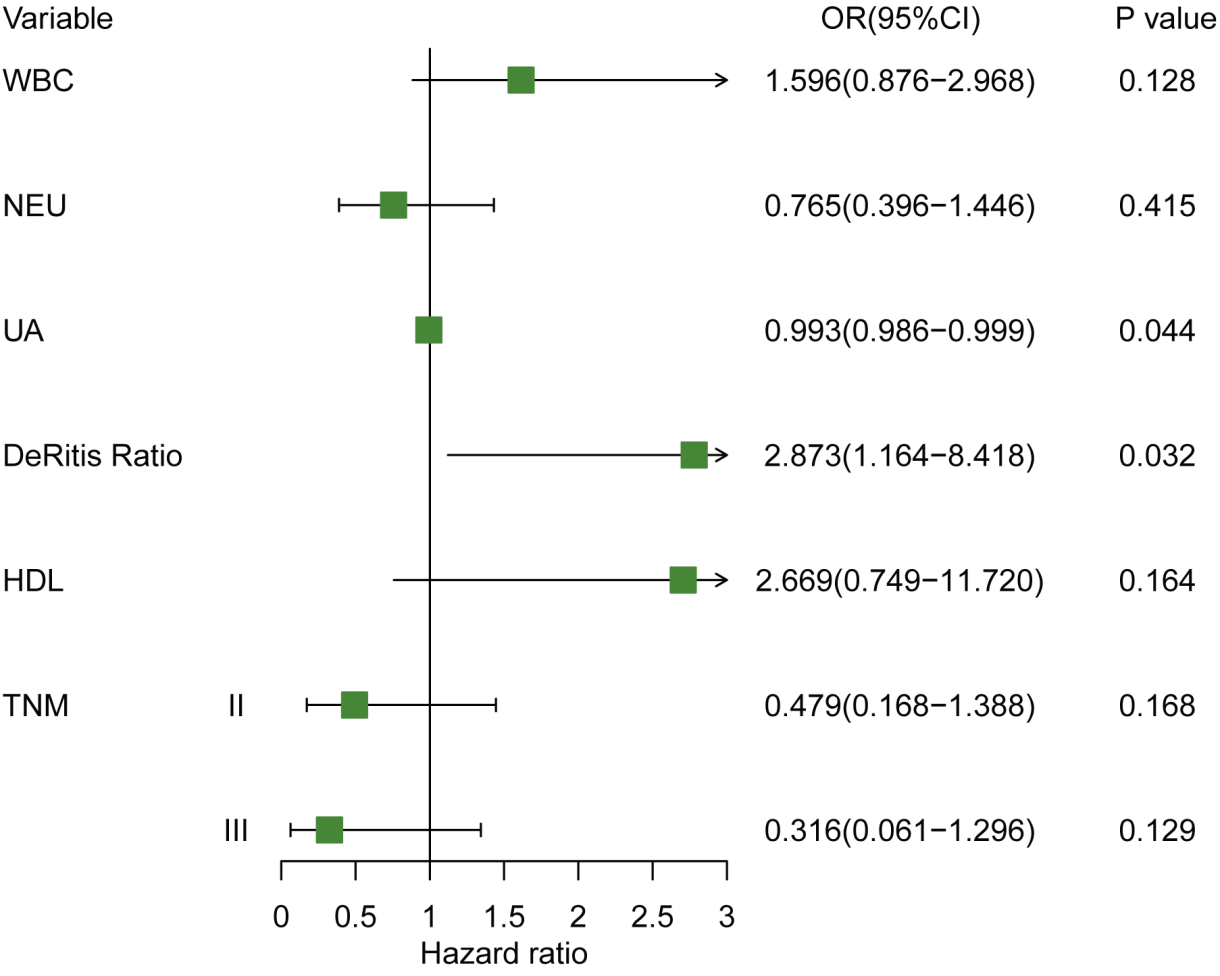
Forest plot of hazard ratio for cHER2 status in tHER2- breast cancer patient. Note: WBC (×10^9^/L); NEUs (×10^9^/L), PLT (×10^9^/L), UA (µmol/L).

To identify the independent predictors for cHER2+ in tHER2- patients, univariable and multivariable logistic regression analyses were used. As shown in **Table 3**, no significant differences were observed in clinicopathological variables between patients with HER2+ CTCs and those without HER2+ CTCs other than TNM stage (T2: odds ratio (OR) = 0.411, 95% CI = 0.162–1.050; *p* = 0.060; T3: OR = 0.234, 95% CI = 0.049–0.850; *p* = 0.040) by univariable logistic regression analyses. In blood tests, WBC (OR = 1.345, 95% CI =1.065–1.714; *p* = 0.014), NEU (OR = 1.280, 95% CI =0.995–1.647; p = 0.052), uric acid (UA, OR = 0.994, 95% CI =0.987–0.999; *p* = 0.027), De Ritis ratio (OR = 3.002, 95% CI =1.299–7.815; *p* = 0.014), and high-density lipoprotein (HDL, OR = 2.943; 95% CI = 0.991–9.952; *p* = 0.059) were also found to be associated with cHER2 status in univariable logistic regression analysis. All of the significant variables above were further analyzed by multivariable logistic regression analysis. UA (OR =0.993; 95% CI = 0.986–0.999; *p* = 0.044) and the De Ritis ratio (OR = 2.873; 95% CI = 1.164–8.418; *p* = 0.032) were independent predictive factors of cHER2 status (**Figure 4**).

**Table 3:** Logistic regression analysis based on clinicopathological parameters/blood test variables for cHER2 status.

Variables	Univariate analysis				Multivariate analysis		
	*OR*	95% Cl	*p-value*		*OR*	95% Cl	*p-value*
Age	0.966	0.918–1.012	0.162				
Pathological type Ductal Other	1.000 2.017	0.413–7.844	0.333				
TNM stage I II III	1.000 0.411 0.234	0.162–1.050 0.049–0.850	0.060* 0.040*		1.000 0.479 0.316	0.168–1.388 0.061–1.296	0.168 0.129
Tumor size T1 T2 T3 T4	1.000 1.222 0.640 2.000	0.123–12.105 0.066–6.223 0.150–26.734	0.864 0.701 0.600				
Nodal status N0 N1 N2 N3	1.000 0.763 0.880 0.347	0.270–1.974 0.185–3.147 0.018–1.990	0.588 0.855 0.327				
ER status Negative Positive	1.000 3.289	0.894–21.299	0.121				
PR status Negative Positive	1.000 0.547	0.224 -1.406	0.194				
Ki67	0.344	0.033–2.536	0.327				
WBC	1.345	1.065–1.714	0.014*		1.596	0.876–2.968	0.128
LY	1.063	0.466–2.322	0.881				
MONO	3.387	0.307–34.650	0.302				
NEU	1.280	0.995–1.647	0.052*		0.765	0.396–1.446	0.415
PLT	1.003	0.997–1.008	0.368				
UA	0.994	0.987–0.999	0.027*		0.993	0.986–0.999	0.044*
CREA	0.991	0.940–1.042	0.724				
UREA	1.009	0.995–1.026	0.203				
De Ritis ratio (AST/ALT)	3.002	1.299–7.815	0.014*		2.873	1.164–8.418	0.032*
TG	0.875	0.474–1.215	0.564				
TC	1.031	0.801–1.262	0.781				
LDL	1.009	0.993–1.027	0.226				
HDL	2.943	0.991–9.952	0.059*		2.669	0.749–11.720	0.164

WBC (×10^9^/L); LY (×10^9^/L); MONO (×10^9^/L); NEUs (×10^9^/L), PLT (×10^9^/L), TG (mmol/L), TC, LDL (mmol/L), HDL (mmol/L), UA (µmol/L), CREA (µmol/L), UREA (mmol/L).

### Predictive nomogram for cHER2+ in tHER2-

**Figure 5:**
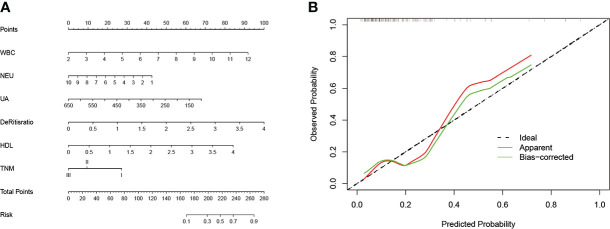
**(A)** The nomogram predicting cHER2 statue in patients with HER2- primary tumor. **(B)** Calibration curve for predicting cHER2 statue in patients with HER2- primary tumor. Note: WBC (×10^9^/L); NEUs (×10^9^/L), PLT (×10^9^/L), UA (µmol/L).

A prognostic nomogram incorporating WBC, NEU, UA, De Ritis ratio, HDL, and TNM stage was developed to predict the cHER2 status in tHER2- patients based on the results above (**Figure 5A**). The HER2+ CTC probabilities can be calculated easily by adding all the scores related to each factor and projecting the total score to the bottom scales.

To evaluate the discriminatory ability of the nomogram, various methods, including C-index values and calibration curves, were used. The C-index demonstrated that the nomogram had favorable predictive accuracy with a value of 0.745 (95% CI: 0.630–0.860). In addition, the relationship between the observed probability and predicted probability can be reflected intuitively *via* a calibration curve. As shown in **Figure 5B**, the calibration curve showed credible consistency between risk prediction and actual results for cHER2 status in tHER2- patients without obvious deviation from the refline.

### Survival analysis of cHER2 status in tHER2- patients

**Figure 6:**
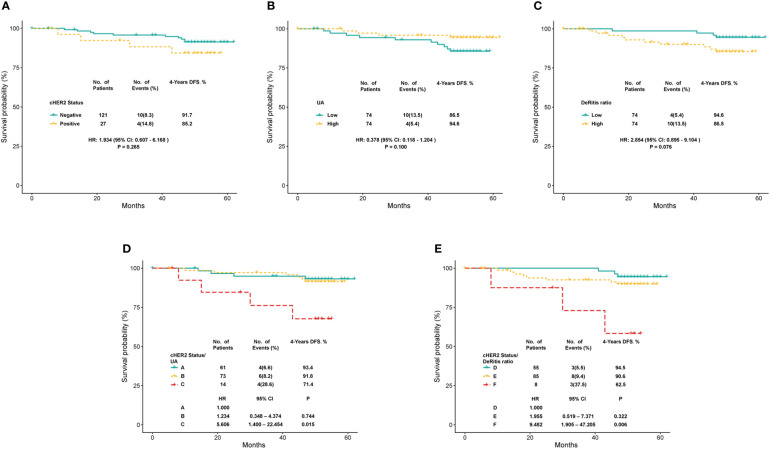
Kaplan–Meier survival curve for 4-year disease-free survival in breast cancer patients with HER2- primary tumor, stratified by **(A)** cHER2 status, **(B)** UA, **(C)** De Ritis ratio, and **(D)** combination of cHER2 status and UA (A: without cHER2+/UA-high, B: without cHER2+/UA-low or with cHER2+/UA-high, and C: with cHER2+/UA-low), **(E)** combination of cHER2 status and De Ritis ratio (D: without cHER2+/De Ritis ratio -low, E: without cHER2+/De Ritis ratio - high or with cHER2+/De Ritis ratio -low, and F: with cHER2+/De Ritis ratio - high).

Survival curves were plotted to show the prognostic efficiency of cHER2 status directly based on 148 tHER2- patients by the Kaplan–Meier method. We found a clear tendency that patients with HER2+ CTCs exhibited much better prognosis, although no significance was reached (*p* = 0.265). Moreover, an HR of 1.934 (95% CI = 0.607–6.168) for HER2+ CTCs was also found by cox regression analysis, which means tHER2- patients with HER2+ CTCs are nearly two times to relapse/recur within 4 years (**Figure 6A**). As UA and De Ritis ratio were found to be associated with cHER2 status independently, as well as the prognostic effect of cHER2 status found by us and others (12–15), the survival effects of these factors were also studied. An obvious trend of better prognosis in patients with a higher level of UA (HR = 0.378, 95% CI = 0.118–1.204, *p* = 0.100) and lower De Ritis ratio (HR = 2.854, 95% CI = 0.895–9.104, *p* = 0.076) was found, although there was no statistical significance (**Figures 6B, C** **)**.

HER2 status of CTCs was combined with the UA/De Ritis ratio to evaluate the possibility of additional prognostic effects. Three different combinations of cHER2 status/UA (A: without cHER2+/UA-high, B: without cHER2+/UA-low or with cHER2+/UA-high, and C: with cHER2+/UA-low) and three of cHER2 status/De Ritis ratio (D: without cHER2+/De Ritis ratio -low, E: without cHER2+/De Ritis ratio - high or with cHER2+/De Ritis ratio -low, and F: with cHER2+/De Ritis ratio - high) were applied for the analysis. Significantly decreased disease-free duration was observed in patients of the subgroup “with cHER2+/UA-low” (C: HR = 5.606, 95% CI = 1.400 – 22.454, *p* = 0.015) and “with cHER2+/De Ritis ratio – high” (F: HR = 9.482, 95% CI = 1.905 – 47.205, *p* = 0.006). No statistical significance was found between subgroups A and B (B: HR = 1.234, 95% CI = 0.348–4.374, *p* = 0.744), as well as D and E (E: HR = 1.955, 95% CI = 0.519–7.371, *p* = 0.322), but a clear tendency of longer disease-free duration was observed in patients of subgroup D when compared with subgroup E (**Figures 6D, E** **)**.

## Discussion

As a potential surrogate marker for monitoring tumor progression and therapeutic target, the prognostic impact and therapeutic value of HER2+ CTCs were studied by many researchers (12–18). Patients with HER2+ CTCs, even those with HER2- primary tumors, appear to benefit from HER2-targeted therapy (12, 16–18), although controversial results were found in the prognostic impact of HER2+ CTCs (12–15). In the present study, we constructed a clinical model to predict the HER2 status in tHER2- patients and survival analysis was also conducted based on the cHER2 status of tHER2- patients.

Currently, the application of HER2-targeted therapy mostly depended on the HER2 status of the primary tumor; thus, tHER2- patients usually do not receive Herceptin (2). Since the detection of HER2+ CTCs in patients with HER2- primary tumor, it raised the question on whether these patients may benefit from the HER2-targeted therapy (17, 18). Researchers have found the successful application of Herceptin in these groups of patients (12, 16–18). The study by Meng et al. found that after nine breast cancer patients with tHER2−/cHER2+ received Herceptin-containing schedules, three achieved partial or complete response (16); in another study by Wang et al., tHER2- patients with a higher number of HER2+ CTC may get more benefit from the HER2-targeted therapy, who achieved a significantly improved progression-free survival (12). Thus, a clinical model to predict tHER2- patients’ cHER2 status is warranted, since the detection of cHER2 status was limited because of the cost and technology; it could help to stratify the tHER2- patients and detect the cHER2 status.

For the ability to predict the possibility of a clinical outcome accurately and conveniently, nomograms have been constructed to assist clinicians in predicting the therapeutic response, recurrence/metastasis, and prognosis in patients with malignancy. Here, we constructed a predictive nomogram based on logistic analysis for predicting the presence of HER2+ CTCs in tHER- patients. To improve the discriminative performance, statistically significant factors, including TNM stage, WBCs, NEUs, UA, De Ritis ratio, and HDL, found in univariate logistic analysis were incorporated, and the performance was assessed by calibration and discrimination. Calibration is defined as the ability to assess the agreement between the predictive risk and the actual risk of the clinical model. In the present study, the calibration plot showed credible agreement in the nomogram predicting the presence of HER2+ CTCs, which suggested the reliability of the nomogram we constructed. Discrimination is the ability to discriminate between patients who experience an event and those who do not (23). The discrimination of a nomogram was evaluated by the C-index. In the current study, the C-index of our model for predicting cHER2 status was 0.745 (95% CI: 0.630–0.860). According to previous studies, a nomogram with a C-index >0.7 was regarded as having an acceptable sensitivity and specificity (24, 25). The relatively small cohort may be the reason for the slightly more extended 95% CI in our model, and further improvement is warranted. Overall, as the first model for predicting cHER2 status, as far as we know, with acceptable discriminative performance, we hope that it can assist clinicians identify early tHER2- patients with a higher risk of developing cHER2+ and aid the treatment strategy decision.

In the present study, HER2+ CTCs were detected in 44 (20.4%) of 216 patients, in which 27 (27/148, 18.2%) patients were tHER2-, and this discrepancy was similar with a previous study (26). In breast cancer patients with progression, *HER-2* gene amplification and HER2+ CTCs could be acquired (27–29). Hayes et al. observed an elevation in CTCs with a very high expression of HER-2 after patients developed clinical recurrence (28). Through an experimental study, HER2 status was found to interconvert spontaneously between cHER2+ and cHER2− *in vitro*, and CTCs of one phenotype can produce daughters of the opposite within four cell doublings (30). However, the mechanism of the alteration was still barely understood, and further mechanistic research is needed.

Our survival analysis based on stage I to III patients found a clear tendency that patients with HER2+ CTCs, a higher De Ritis ratio, and a lower level of blood UA had a much shorter DFS, although they were not statistically significant. HER2-overexpressing epithelial cells in the bone marrow were found to predict poor clinical outcomes of breast cancer patients with/without distant metastasis at diagnosis (31–33); however, the consensus of the prognostic impact of HER2+ CTC in peripheral blood was still yet to be studied (12–15). A study carried out by Wulfing et al. found a significantly decreased DFS and OS in breast cancer patients with HER2+ CTC (34), whereas Wallwiener et al. observed a significantly longer progression-free survival in cHER2+ patients by enrolling 107 participants but showed no difference in OS (15). Therefore, the other risk factors, which may confuse the prognostic effect of HER2+ CTC, should be taken into consideration in the survival analysis. The De Ritis ratio, as a potential prognostic factor for various kinds of malignant tumors, was confirmed to be related to a worse prognosis (35–38). A mechanistic study found that AST plays an important role in glycolysis by relocating NADH into mitochondria through the malic acid–aspartic acid shuttle pathway (39) and thus activates the process of aerobic glycolysis in tumor cells (40). That is, a high De Ritis ratio may reflect the more aggressive behaviors of cancer cells. When concerning UA in malignancy, studies have found its protective role (41, 42). The mechanism of the protective role of UA may be associated with its antioxidative activity by eliminating reactive oxygen species (ROS) (42, 43). ROS are well known to modulate various cell signaling pathways, including MAPK and Ras-ERK signaling, which promote cancer cell proliferation, survival, and migration (44).

By multiple logistic analysis, UA and De Ritis ratio were found to be independent predictors of cHER2 status; we evaluated the additional prognostic effects by incorporating the cHER2 status with UA/De Ritis ratio. Patients of the subgroups “with cHER2+/UA-low” and “with cHER2+/De Ritis ratio – high” had had a significantly worse prognosis. Thus, as we discussed above, we can come to a conclusion with caution that UA and De Ritis ratio confused the prognostic effect of cHER2 status, which may also further validate the results we found in logistic analysis, and corporation with the associated factors could improve the effect of risk stratification.

When interpreting the results, several limitations of the present study should be taken into consideration. First, this was a relatively small group of patients from a single medical center, which may lead to bias. Therefore, trials must be carried out in larger groups in multiple centers. Second, as a clinical trial conducted during a short period, data of OS were lacking. The longer follow-up of patients are warranted in further study. Third, the present study was an observational study; the causality between the risk factors we found and cHER2 status was not clear. Experimental studies in the future are needed to explore these relationships. Fourth, the detection of HER2+ cells is based on cytokeratin-labeled CTCs, and more aggressive CTC subsets are likely to downregulate epithelial biomarkers (45). Thus, the number of HER2+ CTCs was underestimated. In addition, the CTCs isolated in the present study were non-viable and could not be revived for further culture or mechanistic analysis, and new methods are needed to isolate viable CTCs from blood and should be used in further studies.

## Conclusion

In this study, we constructed a predictive nomogram that can determine the cHER2 status of tHER2- early breast cancer patients with clinicopathologic parameters and blood test variables. The model can aid clinicians in the development of more personalized treatment strategies based on the results. Patients who were previously excluded because CTC detection was not a routine test may get great benefit from the application of anti-HER2 targeted therapies. Moreover, our study also found that incorporation with UA and De Ritis ratio may enhance the prognostic value of cHER2 status.

## Data availability statement

The original contributions presented in the study are included in the article/supplementary material. Further inquiries can be directed to the corresponding authors.

## Ethics statement

The studies involving human participants were reviewed and approved by Institutional Review Board of Zhuhai Maternity and Child Health Hospital. The patients/participants provided their written informed consent to participate in this study.

## Author contributions

HZ and WC conceived and designed the experiments. YY, LL, WT, ZQ, QQ, LS and PL conducted and analyzed the data. YY, LL and WT wrote this manuscript. All authors contributed to the article and approved the submitted version.
